# Study comparing the tribological behavior of propylene glycol and water dispersed with graphene nanopowder

**DOI:** 10.1038/s41598-023-29349-7

**Published:** 2023-02-10

**Authors:** A. Haribabu, Raviteja Surakasi, P. Thimothy, Mohammad Amir Khan, Nadeem A. Khan, Sasan Zahmatkesh

**Affiliations:** 1 Department of Mechanical Engineering, Lendi Institute of Engineering and Technology, Jonnada, Vizianagaram, Andhra Pradesh India; 2 Department of Civil Engineering, Galgotia College of Engineering, Knowledge Park I, Greater Noida, Uttar Pradesh 201310 India; 3 Department of Civil Engineering, Mewat Engineering College, Nuh, Haryana, 122107 India; 4 Tecnologico de Monterry, Escuela de Ingenieriay Ciencias, Puebla, Mexico

**Keywords:** Environmental sciences, Environmental social sciences

## Abstract

Nanofluids made up of propylene glycol, and water and graphene nanopowder dispersed throughout them are the primary focus of our study. Nanofluids were created by mixing propylene glycol and water in quantities of 100:0, 75:25, and 50:50. The essential fluids used in this experiment were propylene glycol and water. Graphene was dispersed in these three different base fluids at percentages of 0.25 and 0.5, respectively. This body of work's fundamental objective is to explore nanofluids' tribological behavior. This behavior was observed with a pin-on-disc device, and the impact for load on wear, coefficient of friction, and frictional force was investigated. The tests were conducted with weights ranging from 1 to 3 kg. It was revealed that as the load ascended, there was a reduction in the amount of wear, the coefficient of friction, and the frictional force for the most of the samples tested. Still, there was an increase in the amount of wear and friction coefficient, including the frictional force for some of the samples.

## Introduction

Nanotechnology is an invention of the twenty-first century that has the potential to have a substantial influence on the performance of materials. There has been the development of an alternative nanoscale additive for coolants. In the realm of nanotechnology, one of the most recent and innovative developments is the production of nanofluids. Colloids of nanometric materials suspended in liquids such as water, ethylene-propylene glycol, propylene glycol, or oil are referred to as nanofluids. A particle is a little entity that behaves and moves the same way as a complete item and shares its features. On the other hand, ultrafine particles are defined as having a size that ranges from 1 to 100 nm. Nanoparticles could, but it's not a given that they will exhibit size-linked properties that are noticeably different from those of tiny particles or complete materials. Recent years have seen an unprecedented level of interest in nanoparticle research from the fields of mechanical engineering, biological sciences, and electrical engineering^1–3^.

Many different kinds of applications over the period of the last few decades have demonstrated that nanofluids provide considerable advantages, and this trend is expected to continue. There is a possibility that the use of certain materials could increase the quantity of heat that is efficiently transported by industrial gear. The fundamental goal of nanofluid research has, from the very beginning, not been to decrease the viscosity of nanofluids but rather to increase their thermal conductivity and their ability to transmit heat^4,5^. A nanofluid is a fluid that consists of a base fluid that contains one or more types of nanoparticles scattered throughout the fluid^6,7^. Nanofluids are preferable to microfluidics due to their higher thermal conductivity and lower viscosity when it comes to applications that also include heat transfer. This is due to the fact that nanofluids perform their functions more effectively than microfluidic. Since the 1990s, researchers have investigated the possibilities of enhancing heat transfer methods using nanomaterials to get superior outcomes. They devoted most of their efforts to research associated with creating exceptionally effective procedures for heat transfer technology. Nanofluids have been the focus of research in the fields of material science, physics, and chemistry, due to their enormous application potential in various fields, such as chemical and electrical engineering, and vehicle as building construction, microelectronics, and knowledge. Nanoparticles increase cutting fluid thermal conductivity and tribology. Thermally conducting cutting fluids remove cutting zone temperatures better. Tribological features also facilitate oil film formation at tool-chip and tool-work piece contacts. By preventing the cutting temperature from spreading to the tool and workpiece, cutting force, surface quality, tool wear, and tool life may be significantly improved. Nanoparticle additions minimize friction coefficient, energy consumption, and thermal stress to increase tool life and enhance component surface quality.This is because nanofluids can be used in all of these areas. This is because recent years have seen a significant increase in attention placed on researching nanofluids within materials science, physics, and chemistry. In the past, numerous different mixtures of oxide nanoparticle nanoparticles, such as CuO, Al_2_O_3_, TiO_2_, and Fe_3_O_4_, have been utilized to make nanofluids and evaluate the transport capabilities of such nanofluids. These mixtures of metallic oxide nanoparticles have proven effective in both endeavors^8^.

## Materials and methodology

Propylene glycol, used in these formulations as the fundamental liquid component, was sourced from Naveen Chem. According to the International Union of Pure and Applied Chemistry (IUPAC), propylene glycol is a viscous liquid that is colorless and has a flavor that may be described as slightly pleasing. Another name for propylene glycol is propane-1,2-diol. This substance may be represented by the chemical formula CH_3_CH(OH)CH_2_OH. Because it has two different alcoholic functional groups, it is classified as a diol^9,10^. It may be dissolved in a wide range of solvents, including water, acetone, and chloroform. In their natural state, glycols do not irritate and have minimal volatility. Propylene glycol has several applications, such as creating polymers, the food and beverage industry, and the medical field. The properties of propylene glycol are shown in Table 1.

**Table 1:** Properties of propylene glycol.

Properties	
Chemical formula	C_3_H_8_O_2_
Molar mass	76.095 g·mol^−1^
Appearance	Colorless liquid
Odor	Odorless
Density	1.036 g/cm^3^
Melting point	− 59 °C
Boiling point	188.2 °C
Thermal conductivity	0.34 W/m K
Viscosity	0.042 Pa·s

Graphene is a kind of carbon nanostructure made up of a single sheet of atoms arranged in a honeycomb lattice over two dimensions^11,12^ . This term, derived from the combination of the words "graphite" and the suffix "-ene," describes the tremendous double bonds in graphite. An electron from each atom in a graphene sheet is added to a valence band that spans the whole sheet, and each bit has a strong bond with the three atoms adjacent to it. Carbon nanotubes, PAHs, fullerenes, and even glassy carbon exhibit this kind of bonding to some extent^13,14^. Graphene is a semimetal with remarkable electrical characteristics that may be characterized using theories for massless relativistic particles because the conduction band and the valence band are in touch with one another. These theories can explain graphene's properties. The graphene charge carriers have a linear rather than a quadratic dependence of energy on momentum; it is possible for field-effect transistors made from graphene to achieve bipolar conduction. In addition to exhibiting colossal quantum oscillations and enormous nonlinear diamagnetism, the material demonstrates ballistic charge transfer over great distances.

The plane of graphene is a very efficient conductor of both heat and electricity^15^. Graphite has a considerable capacity to absorb visible light, which is why it has a dark appearance^16,17^. Still, a single layer of graphene is so thin that it is nearly transparent. This compound is almost one hundred times stronger than the most substantial steel compared to the material's strength. Graphene's extraordinary tensile strength, electrical conductivity, transparency, and the fact that it is the world's most robust material have all contributed to its rise in popularity as a valuable and helpful nanomaterial^18^. Graphene is the thinnest two-dimensional substance and the world's most robust material. The research and development sectors of the semiconductor, electronics, electric battery, and composites industries accounted for the vast bulk of graphene's demand in 2012, contributing to the global graphene market's value of $9 million^19,20^. As a result of the hydrophobic property of graphene, graphene oxide (GO) may be readily suspended in a variety of base fluids.

In comparison to traditional carbon nanotubes, graphene possesses superior heat flow properties, which contribute to the material's increased ability to improve the thermal conductivity of nanofluids. Graphene is idealfor other existing nanoparticles in terms of its thermal conductivity, stability, and resistance to erosion and corrosion. Graphene nanopowder was procured from Ultrananotech Pvt.Ltd. Graphene's description as received is shown in Table 2.

**Table 2:** Description of graphene.

Test item	Test result
Purity	> 99%
Thickness	5–10 nm
Length	5–10 microns
Density	3.1 g/cm^3^
Number of layers	Average no. of layers 4–8
Surface area	200–210/g

### Base fluids and nanofluids production

**Figure 1:**
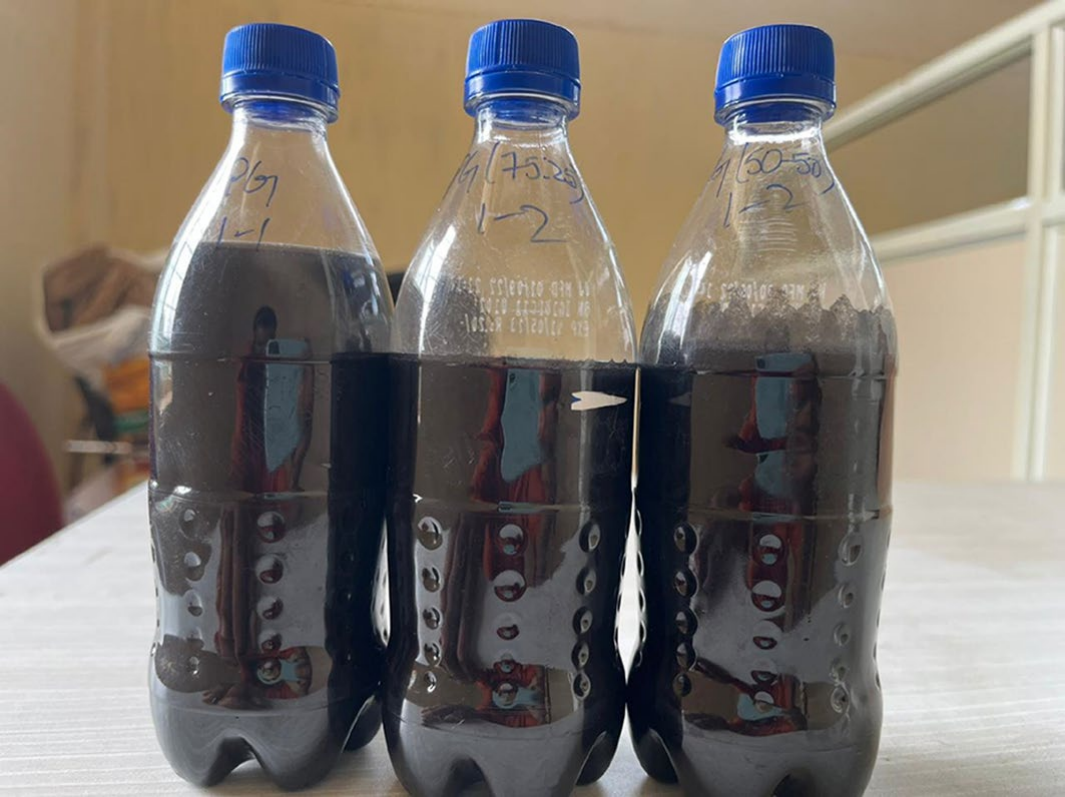
Prepared nanofluid samples.

Propylene glycol–water samples of three kinds (50:50), (75:25), and (100:0) are prepared in this experimentation. The graphene nanoparticles are dispersed in the prepared solutions with the help of an Ultra probe sonicator 0.25 and 0.5 wt%. Figure 1 shows the prepared nanofluid samples.

### Wear

Wear is the process through which a surface gradually deteriorates over time due to erosion or deformation. In addition to mechanical reasons (such as erosion), chemical variables may also contribute to wear and tear (e.g., corrosion). Tribology studies the processes that lead to wear and the factors that contribute to it. Wear, fatigue and creep are three factors contributing to the progressive deterioration of the functional surfaces of machine components. This gradual degradation might ultimately lead to the breakdown of the material or a loss of functionality. It is predicted that abrasive wear negatively impacts between 1 and 4% of the GDP of affluent countries^21^. Metals wear primarily due to the plastic deformation of the surface, near-surface material, and the breaking of particles that generate wear debris. Other factors that contribute to metal wear include heat and friction. The diameters of particles may vary from millimeters down to nanometers^22^. For instance, this may occur when one metal comes into contact with another metal, a nonmetallic solid, a flowing liquid, a solid particle, or a droplet of liquid entrained in a flow of gas^23^. The rate of wear is determined by a variety of different parameters, some of which include the kind of loading (impact, static, or dynamic), the type of motion (sliding, rolling), the operating temperature, and the presence or absence of lubrication^24^. Depending on the tribosystem, it may be possible to identify various kinds and processes of wear. There are a number of tried-and-true methods available for estimating the level of deterioration that has taken place over a certain amount of time and under optimal settings. The ASTM International Committee G-2 is responsible for conducting regular reviews and providing updates to the standards governing wear testing across various sectors and applications. To get a more profound comprehension of tribology, or the study of friction, wear, and lubrication, the Society of Tribologists and Lubrication Engineers has collected data from diverse experiments (STLE). To rate materials in terms of wear resistance, toughness, and other properties, standardized wear tests may be done using the criteria provided in the test's description. Testing in circumstances more indicative of actual use may help improve wear predictions for industrial applications. This is because real-world conditions are more accurate.

### Coefficient of friction

The friction coefficient quantifies the resistance to relative motion between two surfaces. It's a number used in physics to calculate the average force or frictional force exerted by an item when other methods are not available. The formula denotes the coefficient of friction ${F}_{f}= \mu {F}_{n}$ .Within this formula, the frictional force is denoted by F_f_, whereas Fn denotes the normal force. The friction coefficient does not come with any quantifiable units. Because it is scalar, its actual magnitude does not change regardless of the force's direction. The friction coefficient is affected by the many factors that contribute to friction. Although the number is often between 0 and 1, it can be more than 1. The absence of any resistance to motion between two objects is made possible by the superfluidity of the medium. Two things cannot come into contact with one another and not have some reaction to it since friction is required. When the frictional force is equal to one, the normal force is comparable to one. There is a widespread acceptance of the fallacy that the coefficient of friction can only ever be either zero or one. When the coefficient of friction is larger than one, the frictional force, which is measured in comparison to the normal force, is more significant. Silicone rubber is a good illustration of a material with a coefficient of friction much higher than one. The friction force is the resistance to motion produced by a surface whenever an object moves over it or seeks to move over it. This resistance to movement is exerted whenever an item moves over it or attempts to move over it.

### Frictional force

The frictional force is the name given to the force that is generated when two surfaces come into contact with each other as well as slide against one another. Various sources, including the following, cause the frictional force: the surface roughness, as well as the amount of force that's also driving the particles together, are the two most important parameters that play a role in the determination of these forces. On solid surfaces, friction may take the shape of three distinct phenomena: static, sliding, and rolling. The most effective kind of friction is known as static friction, which is then followed by sliding friction, and finally, rolling friction, which is the least effective form. Because gases and liquids are both regarded to be fluids, the phenomenon of fluid friction may take place in either one.

## Experimental procedure

**Figure 2:**
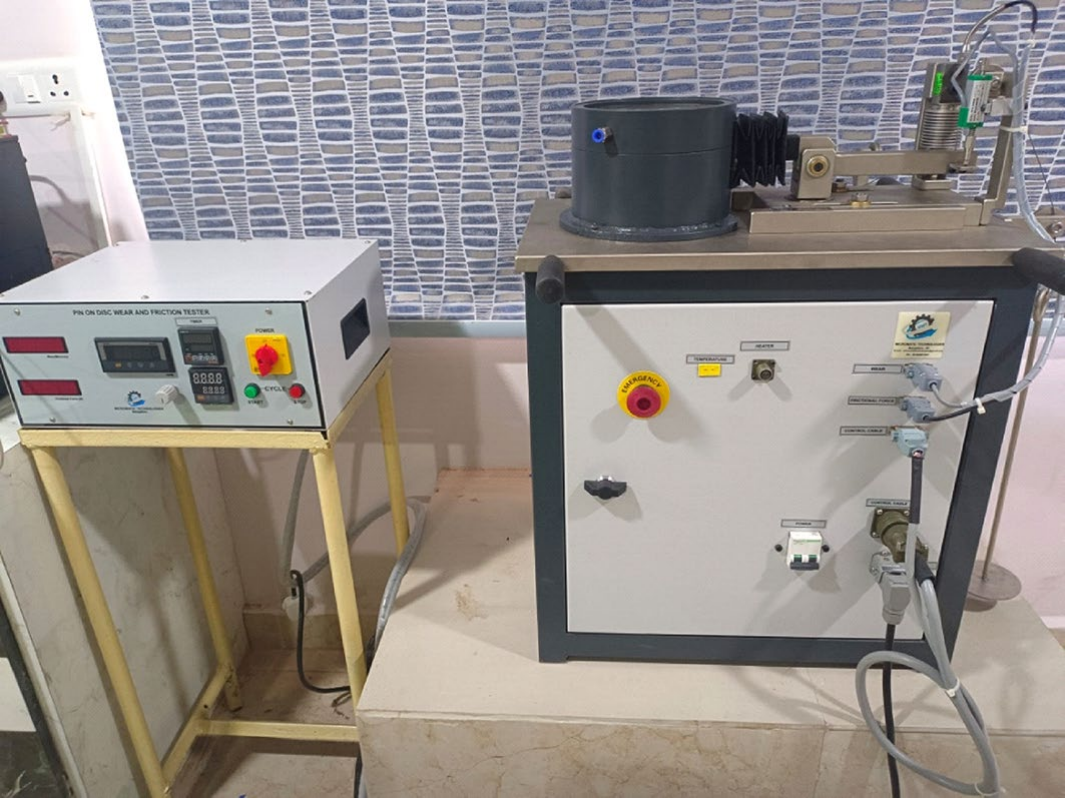
Experimental setup of pin on disc wear testing equipment.

Pin-on-disc instruments assess the wear rate and the frictional force of various materials. The pin-on-disc mechanism consists of a calibrated deadweight-laden pin and a horizontally decaying disc, as shown in Fig. 2. Consider picture 2 for a depiction of the experimental pin disc configuration.

**Figure 3:**
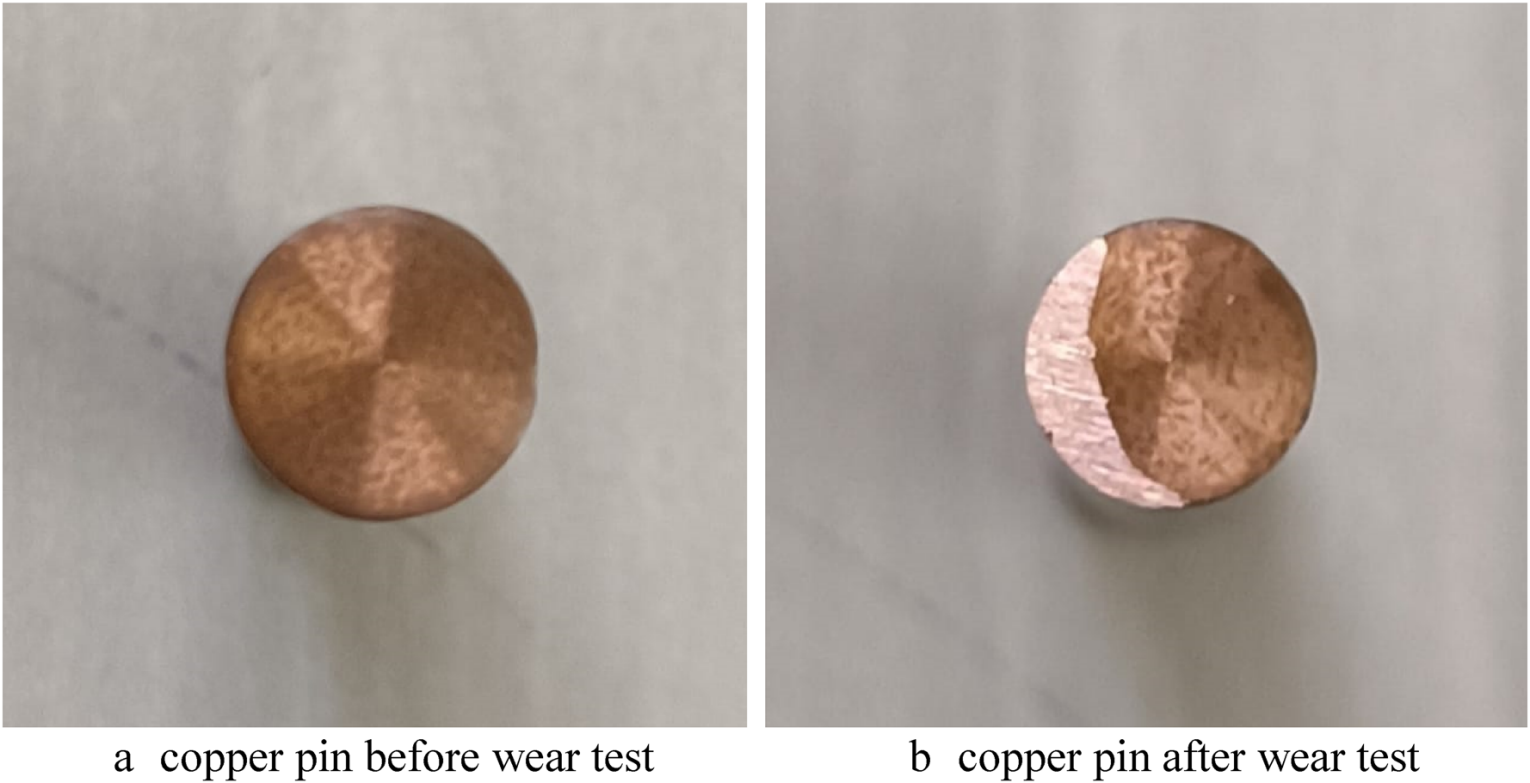
(**a**) Copper pin before wear test, (**b**) copper pin after wear test.

A motor is included to turn the disc.The LVDT sensor is where it shines (Linear variable differential transformers). Wear on the disc occurs when the LVDT sensor's pin touches the disc as it is spinning. Weight is distributed along the lever arm, which carries the pin. The experiments were conducted by varying the load from 1 to 3 kg. A copper pin was used to observe the wear behavior^25^. The Table 3 shows the properties of the pin-on-disc apparatus.Fig. 3 shows the wear property of copper pin before and after conducting the wear test.

**Table 3:** Properties of pin-on-disc apparatus.

Instrument type	Pin on disc
Normal load	10–200 N Dad weights
Sliding speed	0.26–10 m/s
Speed of the disc	50–2000 RPM
Wear measurement range	± 2 mm
Specimen pin diameter	3–12 mm
Specimen standards	ASTM G99 & DIN50324

## Results and discussions

### The effect of load on the rate of wear

**Figure 4:**
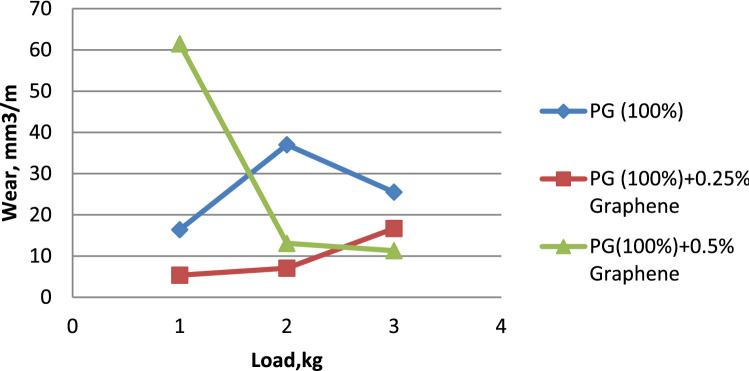
Load vs. wear rate for propylene glycol (100%).

**Figure 5:**
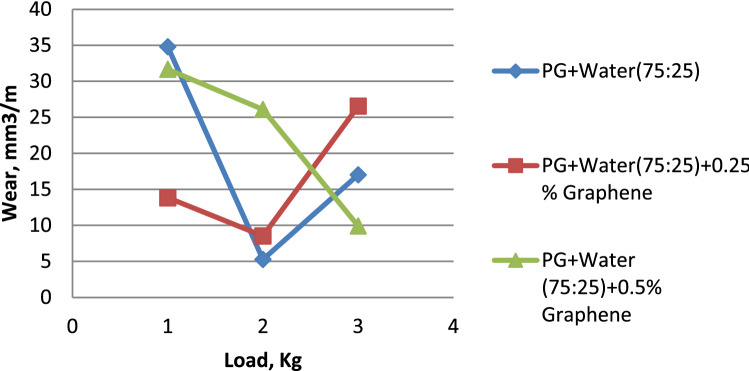
Load vs. wear rate for propylene glycol (75:25).

**Figure 6:**
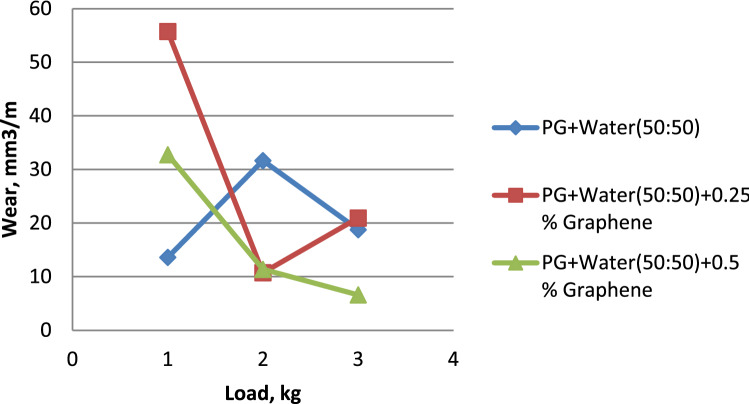
Load vs. wear rate for propylene glycol (50:50).

The influence of load on the wear rate is seen in Fig. 4. In general, wear rises with increasing load variation^26^. However, propylene glycol dispersed with 0.5% graphene exhibits less wear than others. Propylene glycol dispersed with 0.5% graphene shows much less wear than pure propylene glycol and propylene glycol that has been dispersed with 0.25% graphene. It is possible to see in Fig. 5 that Propylene glycol–water (75:25) has a lower wear rate in comparison to Propylene glycol–water (75:25) dispersed with 0.25% Graphene & Propylene glycol–water (75:25) dispersed with 0.5% Graphene. This can be seen in the percentage of graphene dispersed in the solution. When compared to Propylene glycol–water (50:50) along with Propylene glycol–water (50:50) dispersed with 0.25% Graphene, it is clear from Fig. 6 that the Propylene glycol–water (50:50) dispersed with 0.5% Graphene has a lower wear rate. This is because 0.5% graphene has a smaller surface area than 0.25% graphene.From the above results, it can be concluded that the increase in the volume percentage of Graphene decreases the wear rate.

### The influence of load on the friction coefficient

**Figure 7:**
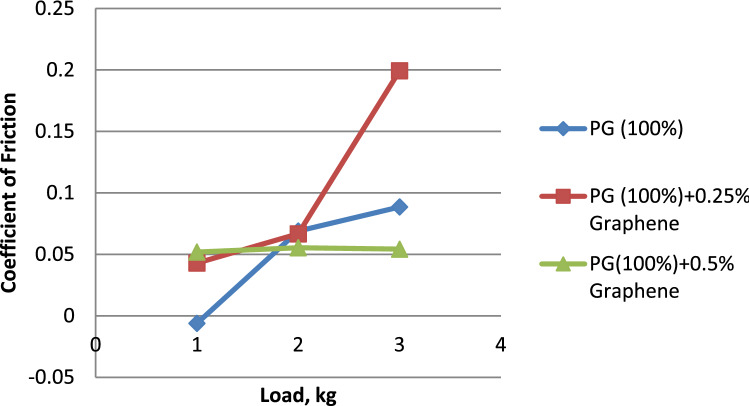
Load vs. coefficient of friction for propylene glycol (100%).

**Figure 8:**
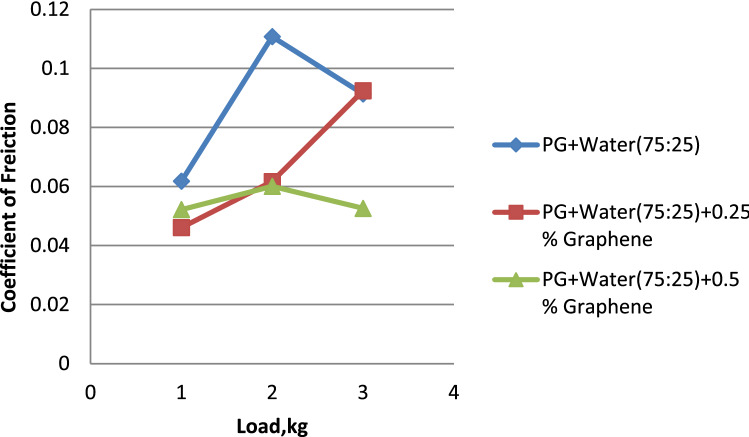
Load vs. coefficient of friction for propylene glycol (75:25).

**Figure 9:**
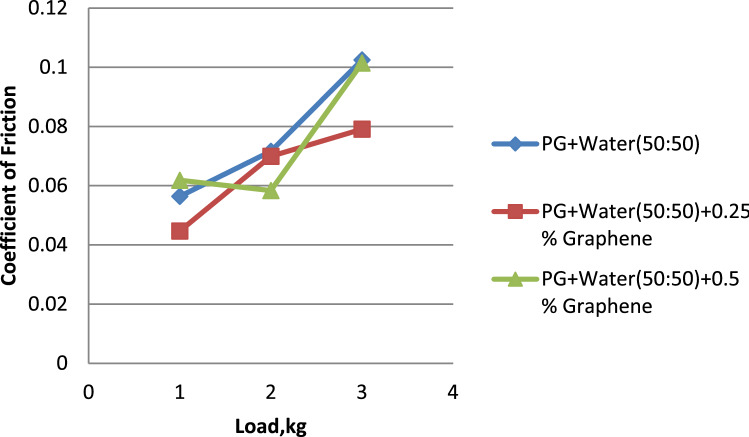
Load vs. coefficient of friction for propylene glycol (50:50).

Figure 8 illustrates the link between the friction coefficient and the load. Figure 7 reveals that the mixture of propylene glycol dispersed with 0.5% graphene has the lowest friction coefficient when compared to pure propylene glycol & propylene glycol disseminated with 0.25% graphene. This is the case when we reach the three formulations. When compared to Propylene glycol–water (75:25) & Propylene glycol–water (75:25) dispersion with 0.25% Graphene in Fig. 8^27^, it is demonstrated that the coefficient of friction is minimum for Propylene glycol–water (75:25) dispersion with 0.5% Graphene. Figure 9 shows that the propylene glycol–water mixture dispersed with 0.25% graphene had the lowest coefficient of friction when compared to the propylene glycol–water mixture dispersed with 0.5% graphene and the propylene glycol–water mixture that was dispersed with 50% graphene^28^. From the above results, it can be concluded that the increase in the volume percentage of graphene. There is a decrease in the coefficient of friction.

### Effect of load on frictional force

**Figure 10:**
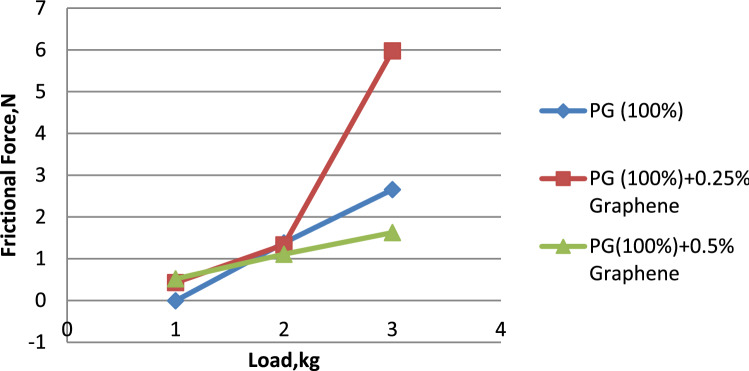
Load vs. frictional force for propylene glycol (100%).

**Figure 11:**
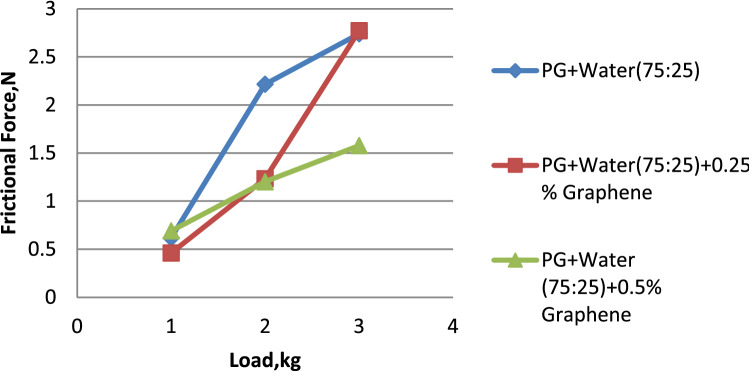
Load vs. frictional force for propylene glycol (75:25).

**Figure 12:**
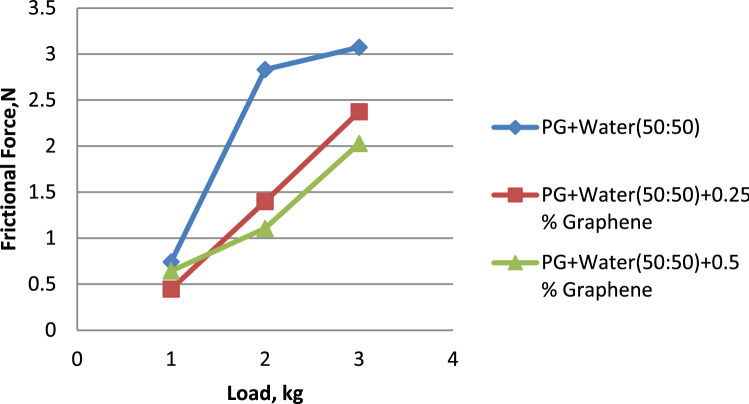
Load vs. frictional force for propylene glycol (50:50).

Figure 10 illustrates the connection between the load and the frictional force. When compared to pure propylene glycol and propylene glycol dispersion with 0.25% graphene, Fig. 10 reveals that the lowest frictional force is seen for propylene glycol dispersed with 0.5% graphene. This is the case even if the total amount of graphene in the mixture is the same. When compared to propylene glycol–water (75:25) and propylene glycol–water (75:25) diffused with 0.25% graphene in Fig. 11, it is clear that the frictional force exerted by propylene glycol–water (75:25) dispersed with 0.5% graphene is significantly lower than that exerted by the other two solutions. Propylene glycol–water (50:50) dispersed with 0.5% graphene was found to have the lowest coefficient of friction in Fig. 12 when compared to both propylene glycol–water (50:50), and propylene glycol–water (50:50) diffused with 0.25% graphene. This finding is compared to the other two formulations^29^. From the above results, it can be concluded that the increase in the volume percentage of graphene decreases the frictional force. Hence graphene was found to be a good alternative for improvement in tribological properties.

## Conclusions

Water and propylene glycol was chosen as the essential fluids, and nanofluids were created by mixing them in quantities of 100:0, 75:25, and 50:50, respectively. In these two kinds of base fluids, graphene was mixed in at 0.25 and 0.5, respectively. 0.5% graphene-dispersed propylene glycol wears less than pure and 0.25% graphene-dispersed propylene glycol. Propylene glycol–water (75:25) showed a lower wear rate than 0.25% and 0.5% graphene-dispersed versions. Propylene glycol–water (50:50) mixed with 0.5% graphene had a lower wear rate than both 50:50 and 0.25% graphene. 0.5% graphene has a smaller surface area than 0.25%. 0.5% graphene-dispersed propylene glycol offers the lowest coefficient of friction. Graphene is included in both formulations. Propylene glycol–water (75:25) mixed with 0.5% graphene had the lowest coefficient of friction compared to both 75:25 and 0.25% graphene. Analysis proves this. Propylene glycol–water (50:50) with 0.25% graphene had the lowest coefficient of friction. This was the case regardless of whether the graphene concentration was 0.5% or 0.25%. Propylene glycol with 0.5% graphene has the lowest frictional force. Propylene glycol–water (75:25) dispersed with 0.5% graphene has a more downward frictional force than the other two. Propylene glycol–water (50:50) with 0.5% graphene had the lowest coefficient of friction.

## Data Availability

The data supporting this study's findings are available from [Raviteja Surakasi]. Still, restrictions apply to the availability of these data, which were used under license for the current research and are not publicly available. However, data are available from the authors upon reasonable request and with permission of [Raviteja Surakasi].
